# xCT (SLC7A11)-mediated metabolic reprogramming promotes non-small cell lung cancer progression

**DOI:** 10.1038/s41388-018-0307-z

**Published:** 2018-05-23

**Authors:** Xiangming Ji, Jun Qian, S. M. Jamshedur Rahman, Peter J. Siska, Yong Zou, Bradford K. Harris, Megan D. Hoeksema, Irina A. Trenary, Chen Heidi, Rosana Eisenberg, Jeffrey C. Rathmell, Jamey D. Young, Pierre P. Massion

**Affiliations:** 1 Cancer Early Detection and Prevention Initiative, Vanderbilt Ingram Cancer Center, Division of Allergy, Pulmonary and Critical Care Medicine, Vanderbilt University School of Medicine, Nashville, TN 37232 USA; 2 Department of Nutrition, Byrdine F. Lewis School of Nursing and Health Professions, Georgia State University, Atlanta, 30302 USA; 3 Department of Internal Medicine III, University Hospital Regensburg, 93053 Regensburg, Germany; 4 Department of Chemical and Biomolecular Engineering, Vanderbilt University, Nashville, USA; 5 Department of Biostatistics, Vanderbilt University School of Medicine, Nashville, TN 37232 USA; 6 Department of Pathology, Microbiology, and Immunology, Vanderbilt University School of Medicine, Nashville, USA; 7 Veterans Affairs, Tennessee Valley Healthcare System, Nashville, TN 37212 USA

## Abstract

Many tumors increase uptake and dependence on glucose, cystine or glutamine. These basic observations on cancer cell metabolism have opened multiple new diagnostic and therapeutic avenues in cancer research. Recent studies demonstrated that smoking could induce the expression of xCT (SLC7A11) in oral cancer cells, suggesting that overexpression of xCT may support lung tumor progression. We hypothesized that overexpression of xCT occurs in lung cancer cells to satisfy the metabolic requirements for growth and survival. Our results demonstrated that 1) xCT was highly expressed at the cytoplasmic membrane in non-small cell lung cancer (NSCLC), 2) the expression of xCT was correlated with advanced stage and predicted a worse 5-year survival, 3) targeting xCT transport activity in xCT overexpressing NSCLC cells with sulfasalazine decreased cell proliferation and invasion in vitro and in vivo and 4) increased dependence on glutamine was observed in xCT overexpressed normal airway epithelial cells. These results suggested that xCT regulate metabolic requirements during lung cancer progression and be a potential therapeutic target in NSCLC.

## Introduction

Although many molecular targets have been identified to improve the treatment strategies in non-small cell lung cancer (NSCLC), 5-year overall survival rate for patients with NSCLC is still 16% [1]. A subgroup of tumors has been found to be driven by genetic alterations in NSCLC, such as EGFR mutations and ALK rearrangements. Tumors with these targetable oncogenic alterations tend to respond to EGFR or ALK inhibitors [2–4]. However, most responders ultimately develop drug resistance and tumor progression. The determinants of tumor progression complicated by the tremendous heterogeneity in molecular alterations in lung cancer are only partially understood. Thus, there is a pressing need for further our understanding of the molecular mechanisms of progression and for the pursuit of innovative therapeutic targets to improve the quality of care and survival of patients with NSCLC.

Recent evidence suggests that metabolic changes, caused by oncogenic activation of signal transduction pathways and transcription factors such as MYC, satisfy the large biosynthetic requirements associated with cancer cell proliferation [5–8]. These metabolic changes include increased glucose consumption, lactate production, and glutamine dependency. xCT (SLC7A11) is a cystine/glutamate antiporter that imports cystine into the cells while exporting glutamate [9, 10]. One molecule of cystine can then be converted into two molecules of cysteine, which is a committed step for glutathione (GSH) biosynthesis. GSH plays a necessary role in maintaining cancer cell function [11]. To quench reactive oxygen species (ROS), GSH is oxidized to GSH disulfide (GSSG), a reaction requiring nicotinamide adenine dinucleotide phosphate (NADPH). Thus, GSH appears as an exciting therapeutic target due to its role in ROS neutralization and detoxification of xenobiotics such as chemotherapeutics. Sulfasalazine (SASP), a FDA-approved drug, has been shown to be functional in the treatment of inflammatory bowel diseases such as rheumatic diseases, Crohn’s disease, and ulcerative colitis [12]. SASP shows inhibitory effects on xCT’s function by decreasing the supply of cystine, an essential step for GSH production [13]. Although high levels of ROS induce cell death and cellular damage, cancer cells tend to maintain a high concentration of GSH to optimize the appropriate redox balance [14]. Targeting xCT may therefore compromise cellular redox defense balance and prevent tumor growth [15].

To maintain the intracellular glutamate pool, cells overexpressing xCT consume more glutamine for glutamate synthesis, a process of glutamine addiction [16]. The dependency on glutamine for cell function is considered a hallmark of cancer metabolism [17]. Different isoforms of glutaminases (GLS), such as GAC and KGA, play major roles in modulating the intracellular glutamine/glutamate concentration [18]. The major function of GLS is to convert glutamine to glutamate with ammonia production. GLS, especially GLS1, is commonly considered as not only a biomarker of glutamine dependence but also a therapeutic target for many types of cancer [19–21]. Recently, higher xCT activity along with elevated intracellular levels of cystine has been shown to promote tumor survival [22] and to contribute to breast cancer progression [16]. Investigators have established the expression pattern of xCT in the NCI 60 cancer cell lines, which suggests that the expression of xCT could act as a predictor of cellular response to chemotherapy [23, 24]. However, the role of this protein has not been studied in details in lung cancer. Therefore, we decided to conduct detailed functional studies to determine whether xCT may cause significant metabolic changes and reprogram the cells for cancer development. The specific goals of this study were: first, to evaluate the expression pattern of xCT protein in different lung cancer subtypes; second, to assess its relevance to the clinical outcomes in NSCLC; and finally, to establish the metabolic functional contribution of xCT in supporting cell growth and viability of lung cancer cells in vitro and in vivo.

## Results

### xCT is overexpressed and is correlated with worse survival in NSCLC patients

**Fig. 1:**
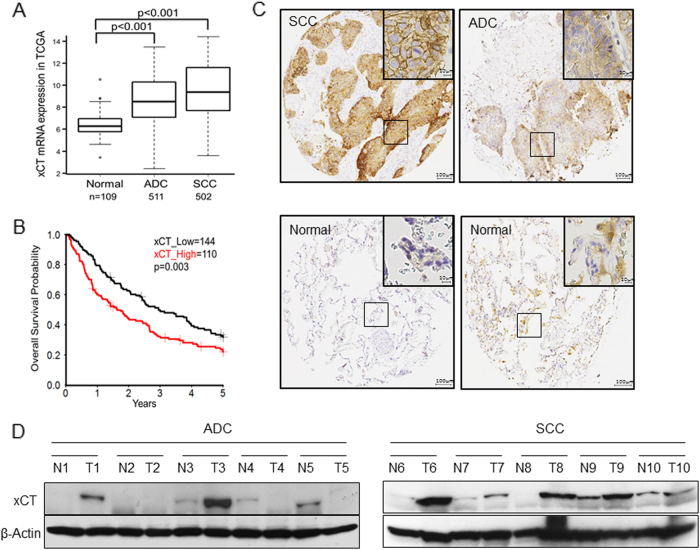
xCT is overexpressed in human NSCLC tumors and predicts worse overall survival. **a** The mRNA expression of xCT is significantly overexpressed in SCC and ADC compared with normal lung tissues in TCGA (*p* < 0.001). **b** Log-rank test demonstrated that the elevated xCT protein expression in TMA is correlated with shorter five-year survival (*p* = 0.003). **c** Representative images of IHC staining for xCT protein, and hematoxylin for nuclei. Top, two representative photomicrographs at ×100 magnification of tumor sections with stage III SCC and III ADC. A zoomed in ×200 magnification of a small area of the same sections in the top right corner showed membranous staining pattern of cancer cells. Bottom, representative photomicrographs at ×100 magnification of normal lung tissue from patients with lung cancer, which stained negative for xCT. **d** Western blots of xCT protein expression from normal human lung (N) and their paired tumor samples (T)

To identify the abundance of xCT expression in NSCLC, we first examined mRNA expression of xCT in tumors including adenocarcinomas (ADC; *N* = 511), squamous cell carcinomas (SCC; *N* = 502) and normal lung tissues (*N* = 109) from The Cancer Genome Atlas (TCGA) database (http://cancergenome.nih.gov/). Our results illustrated that xCT was significantly overexpressed in SCC and ADC compared with normal lung tissues (*p* < 0.0001; Fig. 1a). A cut-off value of mean + 2 SD for xCT in the control group was used to compare the mRNA level of xCT in TCGA lung cancer dataset. With these criteria, we demonstrated that 51% of ADCs (260/511) and 61% of SCC (306/502) were overexpressing the transcripts of xCT.

We then tested the association between xCT protein expression by immunohistochemistry and clinical outcomes in tissue microarrays built in the laboratory and representing a total of 254 NSCLCs (Table 1). Survival analysis using Kaplan-Meier (KM) estimates indicated that elevated xCT expression was correlated with shorter five-year survival rate (*p* = 0.003, Fig. 1b). The pattern of xCT staining in both ADCs and SCCs in the tissue microarrays (TMA) was concentrated along the cytoplasmic membrane, with less intense cytoplasmic staining (Fig. 1c). Further survival analysis using multivariable Cox regression model data showed that elevated expression of xCT protein was significantly associated with worse 5-year overall survival after adjusting for gender, age, smoking history, and stage (*p* = 0.02, Table 2). In addition, we demonstrated overexpression of xCT in seven out of ten primary lung ADCs and SCCs compared to matched normal lung tissues by Western blotting (Fig. 1d and Supplementary Fig. 10A). These data suggest that xCT is overexpressed in NSCLC and the expression of xCT is a potential candidate biomarker in NSCLC.

**Table 1:** Characteristics of patients (n = 254)

Characteristics	Values
Age, mean (SD)	64.81 ± 10.94
Gender	
Male	170 (67%)
Female	84 (33%)
Stage	
I	114 (45%)
II	28 (11%)
III	84 (33%)
IV	28 (11%)
Histology	
Adenocarcinoma	53 (21%)
Squamous cell carcinoma	178 (70%)
Other	23 (9%)
Smoking history	
Current	121 (48%)
Former	120 (47%)
Never	13 (5%)

**Table 2:** Multiple variables Cox proportional hazards analysis for 5-year survival in 254 NSCLC patients

Covariate	HR	95% C.I.		*P*
SLC7A11	1.27	1.04	1.54	0.02
Age	1.01	0.99	1.02	0.48
Gender	1.01	0.73	1.41	0.93
Male vs. Female				
Smoking history				
Former vs. current	0.56	0.4	0.79	0.001
Never vs. current	0.44	0.17	1.09	0.08
Stage				
II vs. I	2.81	1.66	4.76	0.0001
III vs. I	3.87	2.7	5.55	<0.0001
IV vs. I	4	2.43	6.6	<0.0001

### xCT regulates lung cancer cell growth in vitro and in vivo

**Fig. 2:**
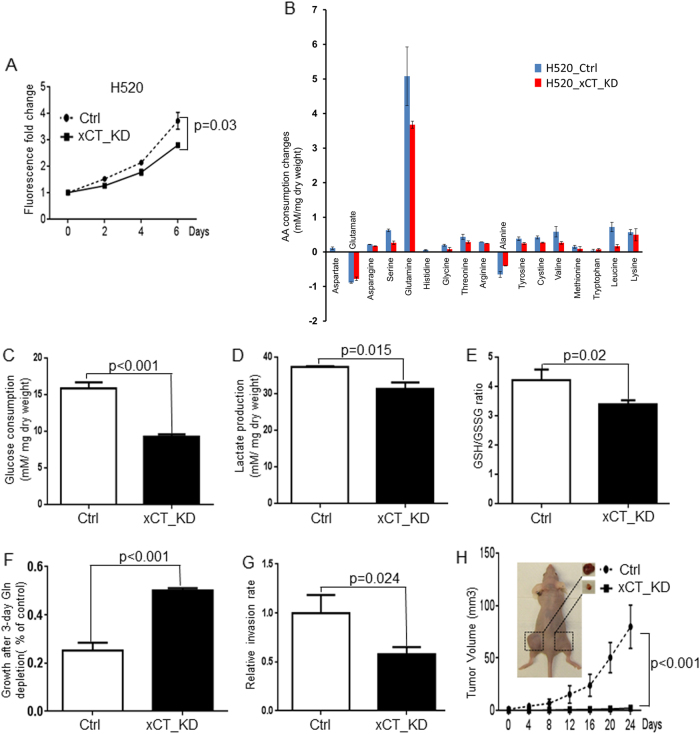
xCT suppression by shRNA inhibits proliferation and tumorigenicity of H520 in vitro and in vivo. **a** Cell proliferation assays reveal significant growth inhibition induced by xCT knockdown in H520 (*n* = 4). **b** Evidence for xCT knockdown in H520 cells to affect the amino acids consumption and excretion in media (*n* = 4). **c** The knockdown of xCT significantly decreases the glucose consumption in H520 cells. **d** The knockdown of xCT significantly decreases the lactate production in H520 cells (*n* = 4). **e** The knockdown of xCT promotes more oxidative intracellular condition in the H520 cells (*n* = 6). **f** The knockdown of xCT significantly decreases the glutamine dependency in H520 cells (*n* = 4). **g** Knockdown of xCT significantly reduces the H520 cell invasion compared with the H520_Ctrl by matrigel cell invasion assay (*n* = 3). **h** The effect of xCT knockdown on tumorigenicity in nude mice. Both H520_Ctrl (left flank) and H520_xCT_KD (right flank) cells were injected subcutaneously into the nude mice (n = 10). Tumor burden was monitored twice a week by caliper. Tumor size was calculated as 3.14 × (Min)^2^ × (Max)/6, where Min and Max were from (length, width, and depth of tumor measurements). All the tumors (mm^3^) were measured in both flanks of 10 mice

To explore the role of xCT in lung cancer development, we silenced protein expression in four xCT overexpressed NSCLC cell lines by shRNA (H520, A549, HCC15, and HCC95, Supplementary Fig. 1A). Consistent with previous results [25], we observed that silencing of xCT inhibited cell growth in four xCT overexpressed cell lines compared with their controls (Fig. 2a and Supplementary Fig. 1B, C and D). Given that xCT is the major cystine transporter, we asked whether knockdown of xCT can reduce cystine and other amino acids consumption. As shown in Fig. 2b, knockdown of xCT reduced the consumption of cystine and release of glutamate in H520 cells. Interestingly, we found that knockdown of xCT also inhibited the consumption of several essential amino acids such as leucine, lysine, and valine, as well as non-essential amino acids such as glutamine and serine (Fig. 2b). Consistent with the hypothesis that xCT mediates the parallel transportation of cystine and glutamate, we observed that knockdown of xCT lowers the secretion glutamate in H520 (Fig. 2b and Supplementary Fig. 5A). We also demonstrated that knockdown of xCT significantly reduced glucose consumption and lactate production in H520 (Fig. 2c,d), A549, and HCC95 cell lines (Supplementary Fig. 3A and Supplementary Fig. 3B) compared with their respective controls. Given that xCT is known to maintain the cellular redox balance by controlling the intracellular glutathione concentration, we asked whether knockdown of xCT could affect glutathione levels. As shown in Fig. 2e and Supplementary Fig. 3C, we found that overexpression of xCT lowered the GSH/GSSG ratio, which created a more oxidative intracellular microenvironment in A549, H520, HCC15, and HCC95 cells. The suppression of xCT expression significantly reduced the glutamine dependency in H520 cells (Fig. 2f), HCC15 cells (Supplementary Fig. 2C) and HCC95 cells (Supplementary Fig. 2D). In addition, xCT_KD cells exhibited impaired invasion in H520, A549, HCC15, and HCC95 (Fig. 2g and Supplementary Fig. 1E, 1F, 1G). We also found that the suppression of xCT expression caused significant inhibition of anchorage-independent colony formation in H520 and A549 cells compared with their control (Supplementary Fig. 1H and Supplementary Fig. 1I).

Based on the results obtained in vitro, we sought to understand the role of xCT in tumor progression in vivo by injecting H520_Ctrl and H520_xCT_KD subcutaneously in the flanks of nude mice. We observed after 24 days that the tumor sizes of all H520_xCT_KD injected mice were significantly smaller than the control group (*p* < 0.001; Fig. 2h). Immunostaining analyses revealed the downregulated expression of xCT and Ki 67 protein in H520_xCT_KD cells (Supplementary Figs. 2A and 2B). Taken together, these results suggested that xCT is essential for the growth and development of NSCLC cells in vitro and in vivo, and knockdown of xCT reduces the cell invasion and glutamine dependency in NSCLC.

### Targeting xCT function attenuates tumor growth in vitro and in vivo

**Fig. 3:**
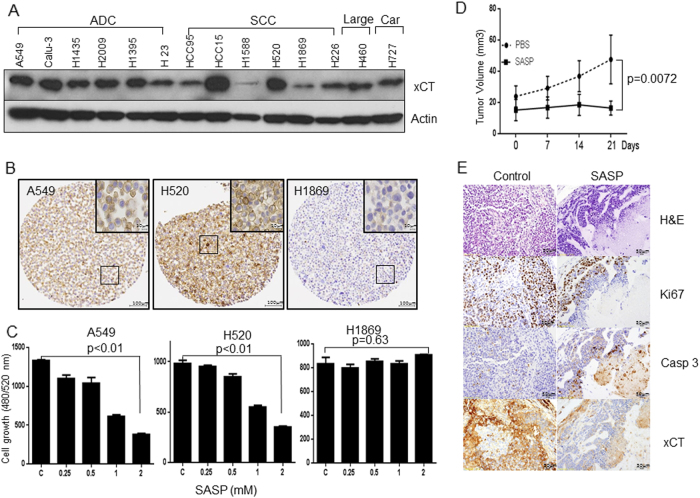
Targeting xCT in NSCLC by SASP. **a** xCT is highly expressed among different types of lung cancer cell lines. **b** Representative images of IHC staining for xCT protein in NSCLC cells, A549, H520, and H1869. **c** Cell proliferation assays reveals dose-dependent growth inhibition induced by SASP in A549 and H520 but not in H1869 NSCLC cells (*n* = 4). **d** Treatment of SASP reduces the tumor size in nude mice. Five million H520 cells were implanted into flanks of nude mice (*n* = 10). After two weeks, mice were randomly assigned into two groups. Tumor burden was monitored twice a week by caliper. Tumor size was calculated as 3.14(Min)^2^ × (Max)/6, where Min and Max were from length, width, and depth of tumor measurement. **e** H&E staining, immunohistochemical staining for Ki67, cleaved Caspase 3 and xCT in tumor formed by H520 in SASP treated or control mice

Next, we examined the expression of xCT in a subset of NSCLC cells (Fig. 3a). The expression of xCT in A549, H520, and H1869 cells was confirmed in our cell line microarray (Fig. 3b). We found that SASP inhibited the proliferation of xCT-positive cells lines (A549 and H520) at 72 h after exposure in a dose-dependent manner. In contrast, SASP had little effect on the proliferation of cells expressing low levels of xCT (Fig. 3c). On the basis of these in vitro data, we tried to determine whether targeting xCT by SASP had an anti-tumor effect in xCT-positive cells in vivo. We injected H520 cells into ten nude mice and randomly assigned the mice into two groups treated either with PBS or SASP for three weeks (Supplementary Fig. 2E). As shown in Fig. 3d, mice receiving SASP (200 mg/kg) had significantly smaller tumor size compared with the PBS-injected group. Immunostaining analysis revealed that SASP lowered the expression of xCT and decreased the Ki67 staining compared with control group (Fig. 3e). In addition, we observed a major elevated expression of cleaved Caspase 3 (Fig. 3e and Supplementary Fig. 2F) in the SASP-treated group compared with PBS group. These results provide in vivo evidence for targeting xCT as a potential therapeutic strategy for NSCLC.

### xCT overexpression promotes proliferation and glutamine dependency in normal human airway epithelial cells

**Fig. 4:**
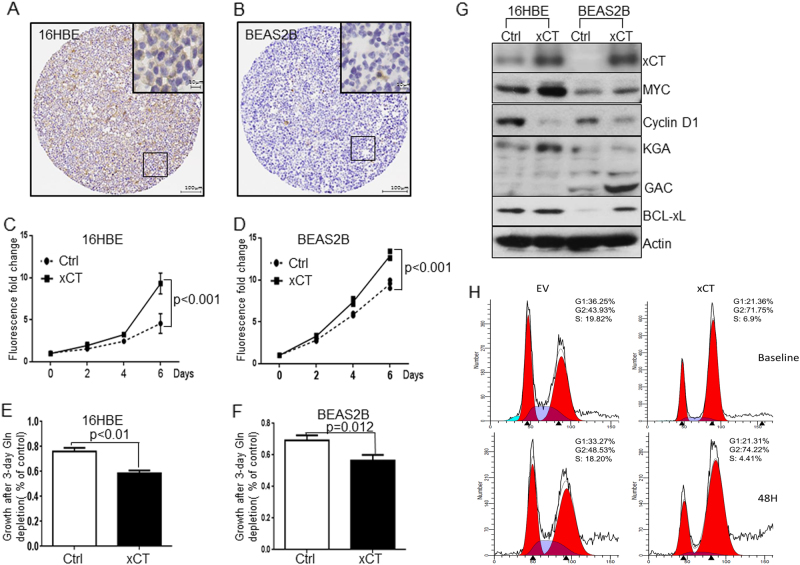
Overexpression of xCT promotes the proliferation and glutamine dependency of human normal airway epithelium cells. **a**, **b** Representative immunohistochemical staining for xCT protein expression in a section of normal airway epithelium cells A (16HBE) and B (BEAS2B). Overexpression of xCT promotes cell proliferation in 16HBE (**c**) and BEAS2B (**d**) (*n* = 4). Glutamine deprivation assays shows that xCT overexpression promotes the glutamine dependency in 16HBE (**e**) and BEAS2B (**f**) in 72 h (*n* = 4). **g** Western blot demonstrates that overexpression of xCT is associated with MYC and its downstream molecular pathways such as GLS1, BCL-xL, and Cyclin D1 overexpression in 16HBE and BEAS2B cells (*n* = 3). **h** Effects of xCT overexpression in BEAS2B cell cycle distribution. Representative flow cytometry profiles and the corresponding ratio of cells in G1, S, and G2 phase at serum starvation baseline and after 48 h (*n* = 3)

To elucidate the mechanisms by which xCT regulates cell proliferation, we first examined its expression in normal airway epithelial cells. As shown in Fig. 4a,b, the protein expression of xCT at baseline was lower in normal airway epithelial cells (16HBEs and BEAS2Bs) compared with the staining of xCT in cancer cells (Fig. 3b). Overexpression of xCT significantly promoted the proliferation of 16HBE (Fig. 4c) and BEAS2B cells (Fig. 4d) as compared with their controls.

Glutamine dependency is a hallmark of cancer development [26], and xCT contributes to glutamine metabolism by depleting the glutamate pool. We, therefore, tested whether cells overexpressing xCT enhanced the cell dependency on glutamine. We performed cell proliferation assays under glutamine deprivation and found that cells overexpressing xCT were more sensitive to glutamine deprivation. This was observed in 16HBE_xCT (Fig. 4e) and BEAS2B_xCT (Fig. 4f) compared with their matched controls. Cancer cells rewired their metabolic pathways such as elevated glucose consumption, lactate production, and glutaminolysis. Glutaminases 1 (KGA and GAC) are the major enzymes that catalyze glutaminolysis by converting glutamine to glutamate. As shown in Fig. 4g, overexpression of xCT promoted the expression of KGA in 16HBE cells and GAC in BEAS2B cells. Previous studies have suggested glutaminases are targets of the MYC pathway which leads to the glutamine dependency [27, 28]. Consistent with these data, we observed increased MYC expression in 16HBE_xCT and BEAS2B_xCT cells compared with their controls. In addition, flow cytometry data demonstrated that overexpression of xCT reduced the ratio of cells in G1 phase, which increased cells in G2 phase (Fig. 4h). Consistent with the previous report [29], cells that arrested in G2 phase became aneuploid as a result of MYC overexpression. This process was known as endoreduplication [30]. Collectively, these results demonstrated that xCT promoted proliferation and glutamine dependency in normal airway epithelial cells.

### xCT overexpression in normal airway epithelial cells induces metabolic reprogramming

**Fig. 5:**
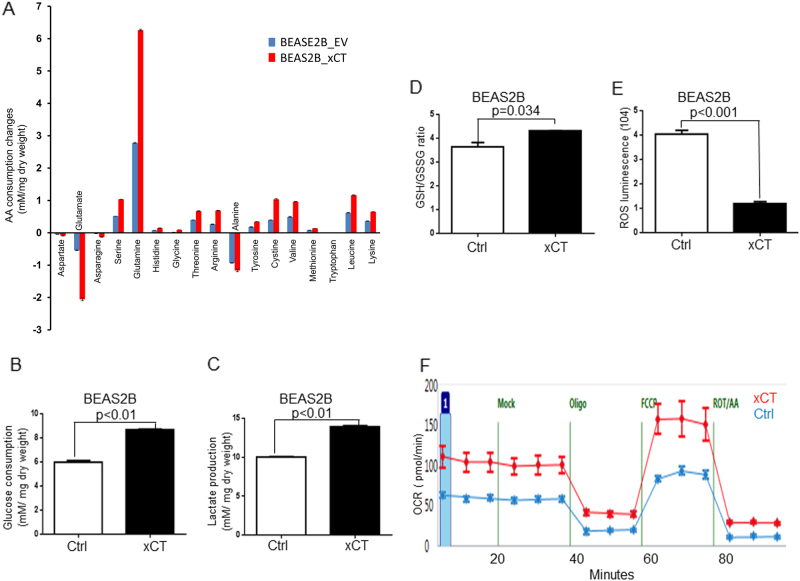
xCT overexpression induces metabolic reprogramming in normal airway epithelial cells. **a** Evidence of xCT overexpression in BEAS2B cells modifies the amino acids consumption and excretion in media (n = 4). The overexpression of xCT increases the glucose consumption (**b**) and lactate production (**c**) (*n* = 4). **d** The overexpression of xCT increases in GSH/GSSG ratio and promotes more reductive intracellular conditions in the BEAS2B cells (*n* = 6). **e** The overexpression of xCT reduces ROS in BEAS2B (*n* = 6). **f** Overexpression of xCT elevates the oxygen consumption rate (OCR) in BEAS2B cells. The plot of OCR showed over time with the addition of oligomycin (1 µM), mitochondrial uncoupler FCCP (1 µM), and electron transport inhibitors antimycin (0.5 mM) + rotenone (0.5 mM). Maximal respiration, proton leak, and coupled respiration were determined as indicated (*n* = 6)

Metabolic reprogramming characterized by elevated glucose consumption, lactate production, and glutamine addiction, was one of the hallmarks of cancer biology [31]. We then asked if increased xCT expression in normal airway epithelial cells would be sufficient to cause metabolic reprogramming and increase the sensitivity to xCT inhibition. Because xCT is the major cystine/glutamate antiporter, we speculated that overexpression of xCT might induce profound metabolic alterations in culture media of BEAS2B_EV and BEAS2B_xCT. As shown in Fig. 5a, the consumption of glutamine and cystine were significantly increased in BEAS2B_xCT cells compared with BEAS2B_EV cells. By measuring the intracellular and extracellular concentration of glutamate, we found overexpression of xCT promotes the glutamate secretion (Fig. 5a and Supplementary Fig. 5B). In addition, we found that SASP more effectively potentiate the cell migration in BEAS2B_xCT compared with the control (Supplementary Fig. 5C). We also found that overexpression of xCT promoted glucose consumption (Fig. 5b), and lactate production (Fig. 5c) in BEAS2B cells. Uptake of a fluorescent labeled glucose analog (2NBDG) confirmed that overexpression of xCT promoted glucose consumption in 16HBE (Supplementary Fig. 4C) and BEAS2B (Supplementary Fig. 4D). Because cystine was reduced to cysteine for glutathione (GSH) biosynthesis in the cytoplasm, we tested GSH production in our system and found that overexpression of xCT increased the GSH/GSSG ratio (Fig. 5d), as well as in 16HBE cells (Supplementary Fig. 4A). Because the function of GSH was to serve as reactive oxidant species (ROS) scavenger, we also investigated changes in ROS concentrations. We found that overexpression of xCT significantly reduced the total amount of ROS in BEAS2B_xCT compared with BEAS2B_EV (Fig. 5e). Similar results were confirmed in 16HBE cells (Supplementary Fig. 4B). In addition, we found overexpression of xCT substantially increased the sensitivity to SASP in two normal airway epithelia cells (Supplementary Fig. 4G). Given that cigarette smoking is one of the major risk factor for lung cancer development, we surmise that cigarette transform the normal cells by the induction of xCT expression. After 72 h treatment, we found that cigarette smoking condensate slightly increased the expression of xCT in BEAS2B cells (Supplementary Fig. 4E). By co-culturing the primary bronchial epithelial cells with irradiated feeder cells, we did not find the significant elevated expression of xCT after 14-days treatment (Supplementary Fig. 4F).

In search for the effect of overexpression of xCT on mitochondrial activity in normal airway epithelial cells, we examined the status of mitochondrial respiration in the BEAS2B_EV and BEAS2B_xCT cells by seahorse analysis. After normalization to cell number, both basal oxygen consumption rate (OCR) and maximal respiratory capacity were significantly elevated after xCT overexpression (Fig. 5f). Maximal respiratory capacity was measured by treating cells with oligomycin (1 uM) to block ATP production. After that, uncoupling agent carbonyl cyanide p-trifluoromethoxyphenylhydrazone (FCCP, 1 uM) was added to dissipate proton gradients which allowed electron transportation and oxygen consumption to operate at the maximal rate. This elevated OCR was suppressed by the electron transport inhibitors, antimycin A and rotenone (0.5 mM, each), showing that respiration was mitochondrial (Fig. 5f). Thus, overexpression of xCT caused metabolic reprogramming in normal airway epithelial cells that was coupled with oxidative phosphorylation.

## Discussion

Accumulating evidence demonstrated that xCT plays an important role in the development and the survival of different cancer cell types including breast, glioma, and lymphoma [16, 32–34]. Here we described the functional significance of xCT overexpression in NSCLC. Specifically, we report that xCT is overexpressed in the cytoplasmic membrane and the overexpression is correlated with poor five-year survival in patients with NSCLCs (Fig. 1). Our results prove that xCT regulates glutamine dependency measured by cell proliferation assay. In order to understand the efficacy of targeting xCT in NSCLC, we chose a panel of cell lines with different expression levels of xCT to pursue functional analysis. Our results reveal that a subgroup of cells with higher xCT expression are particularly sensitive to xCT knockdown, which inhibits cell growth, colony formation in soft agar, and cell invasion (Fig. 2).

SASP has been shown as the potent pharmacological agent to inhibit xCT transport activity in different cancer cell types, including those from the esophagus, breast, and bladder [16, 33, 35]. In addition, SASP was known as a potent inhibitor of xCT in small cell lung cancer cells by depleting glutathione [34]. Consistent with these studies, our results suggested that targeting xCT with SASP significantly inhibit the proliferation of xCT-expressing NSCLC cells, A549 and H520 (Fig. 3). Our *in vitro* data was confirmed *in vivo* by showing that intraperitoneal administration of SASP twice daily for three weeks significantly reduces tumor burden in nude mice. The growth inhibitory effect of SASP on nude mice xenografts is attributed to the induction of apoptosis in xCT overexpressing cells. Thus, the results presented in Fig. 3 together with the correlation between the expression of xCT and 5-year survival provide the rationality for targeting xCT in NSCLC.

The intensity of the immunohistochemical staining for xCT by NSCLC has already been correlated with (4 S)-4-(3-[18 F]fluoropropyl)-l-glutamate uptake [36] making xCT expression a promising candidate biomarker predictive of cystine uptake. Our results show that the xCT expression is not only a good candidate biomarker for diagnosis of lung cancer but also associated with response to sulfasalazine, suggesting the potential pertinence of xCT expression as a novel candidate companion biomarker in NSCLC.

xCT has known metabolic functions in normal and cancer cells as an antiporter of cystine and glutamate [16, 37]. Yet, the specific function of xCT in lung cancer development had not been explored. Therefore, we investigated the contribution of xCT to metabolic reprogramming in airway epithelial cells. Previous in vitro studies reported that xCT expression is elevated at the mRNA level in oral cancer cells upon the exposure to cigarette smoke condensate [38]. Given that smoking is the top risk factor for lung cancer development, we hypothesize that smoking induced expression of xCT in normal airway epithelial cells could be an adaptation mechanism to enable lung cancer development. Our results indicate that overexpression of xCT increases the consumption of many amino acids such as glutamine, cystine, valine, leucine, and lysine. In addition, we also observed that cells overexpressing xCT consume more glucose, glutamine, and produce more lactate with upregulation of oxidative phosphorylation. Interestingly, overexpression of xCT is associated with glutamine dependency in normal airway epithelial cells. In addition, our results demonstrate that overexpression of xCT induces the expression of MYC and decreases ROS production in normal airway epithelium cells. Consistent with previous data, the overexpression of MYC induced by xCT causes cell cycle arrest in G2 [29]. This relaxation of G2 phase may be an essential early step in tumor initiation due to genomic instability [29]. Previous data demonstrated that overexpression of MYC induces the expression of mitochondrial genes and increases ROS production in cancer cells [39]. In order to maintain the integrity of cells, most cancer cells increase their antioxidant level to defend ROS-induced damage. It would be interesting to elucidate the relationship between the overexpression of xCT and role of ROS production during tumor development. Finally, our results suggest that overexpression of xCT play significant role in reprogramming glutamine metabolism and proliferation in lung cancer cells. This proliferative effect induced by xCT can be attributed to a MYC-dependent glutaminolysis pathway [27]. Furthermore, our results suggest that xCT significantly contributes to glutamine catabolism and induces MYC expression in normal airway epithelial cells. The arrest of the G2 cell cycle may be an early step in tumor development.

In conclusion, our results demonstrate that xCT is a major regulator of metabolic reprogramming with overarching effects on glucose metabolism, glutamine dependency, and intracellular GSH/GSSG redox balance. All these metabolic effects contribute to lung cancer development. The expression of xCT is correlated with poor prognosis in NSCLC and represents a novel companion biomarker of a therapeutic target in a molecularly stratified NSCLC patients. Further studies direct toward understanding the cross-talk between xCT and other lung oncogenic pathways (MYC, KRAS, and NOTCH) in the development of tumorigenesis.

## Materials and Methods

### Patients and construction of tissue microarray

Tissue microarray (TMA) was constructed using fixed and paraffin wax–embedded tissues from 254 patients according to protocols previously described [40]. The TMAs consisted of 53 adenocarcinomas, 178 squamous cell carcinomas, and 23 other types of NSCLC. Archived tissue blocks obtained from the pathology departments of the Vanderbilt University Medical Center were stained and reviewed at the pathology department of the Vanderbilt University Medical Center.

To compare the mRNA level of xCT in TCGA lung cancer dataset, a cut-off value was set as a mean + 2 SD for xCT in the control group.

### Cell culture

Total of fourteen human NSCLC cell lines were purchased from the American Type Culture Collection (ATCC) as following: six lung ADC cell lines (A549, Calu-3, H1435, H2009, H1395, and H23), six SCC cell lines (HCC95, HCC15, H1588, H520, H1869, and H226), one large-cell carcinoma cell line (H460), and one carcinoid cell line (H727). In addition, two immortalized lung epithelial cell lines were used in our study. Among them, BEAS2B was purchased from ATCC, and 16HBE was a gift kindly provided by Dr. Dieter Gruenert, UCSF. All cancer cell lines were maintained in RPMI-1640 or DMEM media (Life Technologies, Grand Island, NY). 16HBE and BEAS2B were cultured in DMEM. All cells were grown in 1% penicillin/streptomycin and 5% CO_2_ with 10% fetal bovine serum.

Lentiviral-based stable transfection was performed as previously reported [41] (Dharmacon, Lafayette, CO). We used CCSB-Broad lentiviral expression system to overexpress either human xCT (ccsbBroad304-02826) or its relative vector (Ctrl) in two airway epithelium cells (BEAS2B and 16HBE). After the antibiotic selection (10 µg/ml blasticidin), the expression of xCT was confirmed in 16HBE and BEAS2B cells by Western blotting. On the other hand, we used a pool of shRNA (V2LHS-251161, V3LHS-392254, and V3LHS-392256) (Dharmacon, Lafayette, CO) for xCT knock down in A549, H520, HCC15, and HCC95 cell lines with 1.5 μg/mL puromycin for selection. After selection, pooled cells were tested for xCT expression using Western blotting.

### Proliferation assays

Proliferation assays were performed as previously described [41]. Briefly, cells were plated in 24-well tissue culture plates at 4 × 10^4^ cells/well. Cultures were grown up to 6 days. The direct CyQUANT assay (Life Technologies, Grand Island, NY) was then done according to the manufacturer’s instructions at indicated times up to 6 days. We used a fluorescence microplate reader to record emission wavelength at 520 nm after 480 nm excitation. A representative viability experiment is shown with mean and standard deviation (SD).

### Soft agar colony formation assay

To test the anchorage-independent growth in vitro, we perform soft agar colony formation assay as previous described [41]. The bottom agar contained cell culture growth medium with 0.8% agarose in 6-well plates. H520 with empty vector (H520_Ctrl) and H520 xCT knock down (H520_xCT_KD) cells were plated on top of the agar layer at 20,000/well with DMEM medium containing 10% fetal bovine serum and 0.4% agarose. The cells were incubated at 37 °C in 5% CO_2_ for 30 days. Total colonies were stained with MTT and quantified using a dissecting microscope (Olympus, PA).

### Matrigel cell invasion assay

Matrigel-coated transwell inserts (BD Biosciences, San Jose, CA) with the eight µm-pore membranes were used to test the cell invasive ability of xCT knockdown in A549, H520, HCC15, and HCC95 cells. A total of 5 × 10^4^ Ctrl and xCT_KD cells in basal media were transferred into the upper chamber, and the lower chambers were filled with 400 µL of DMEM with 10% FBS. After 24 h incubation, the cells in the upper chamber were scraped, and adherent cells attached to the lower surface of the insert were incubated with a cell-permeant dye calcein, which can be converted by live-cell esterase to produce green fluorescence and counted.

### Metabolic assays

High-performance liquid chromatography (HPLC, Agilent 1200 series) with a gradient elution method on a reverse-phase column was used to measure amino acid concentrations in the medium [42]. Briefly, media samples were derivatized using orthophthalaldehyde (OPA). After that, ZORBAX Eclipse PLUS C_18_ column (Agilent Technologies, 4.6 × 150 mm, 3.5 μm) was used for amino acid concentration determination. The mobile phases were set up as the following: buffer A: 10 mM Na_2_HPO_4_, 10 mM Na_2_B_4_O_7_, and 8 ppm NaN_3_ in water; buffer B: a mixture of methanol: acetonitrile: water as the ratio of 9:9:2. The gradient elution was: 0–0.5 min, 2% of B; 0.5–15.5 min, linear from 2% to 47% of B; 15.5–15.6 min, linear from %47 to 100% of B; 15.6-19 min hold at 100% of B; 19–19.1 min, linear to 2% of B; and 19.1–21 min,hold at 2% of B. The flow rate was kept at 1.5 mL/min, and the column was maintained at 40 ℃ during the running.

Seahorse mitochondria function assays were normalized to cell number according to the commercial protocol (Agilent Technologies, Santa Clara, CA). The oxygen consumption rate (OCR) was determined with an XF96 extracellular flux analyzer (Seahorse Bioscience) using manufacturer-recommended protocols. For our experiments, OCR was measured over time following injection of final concentrations of oligomycin (1 µM), FCCP (1 μM), and Rotenone/antimycin A (0.5 µM).

Relative concentrations of glutathione (GSH) and oxidative of glutathione (GSSG) were measured by GSH-Glo Glutathione assay according to the protocol (Promega, WI). Briefly, 10,000 cells were seeded and cultured in 96-well plates for 6 h. After that, GSH or GSSG lysis buffer were directly added into the culture wells, and luciferin was added for detection. Luminescence was then read and recorded after background subtraction. Six wells per experimental group were used.

Glucose consumption and lactate production were measured using 2300 STAT Plus dual glucose/lactate analyzer (YSI Life Sciences, Yellow Springs, OH). These data were normalized to the total protein concentration.

Glucose uptake was analyzed after treatment with the fluorescent glucose analog 2-[N-(7-nitrobenz-2-oxa-1, 3-diazol-4-yl) amino]-2-deoxy-D-glucose (2-NBDG) as described previously [43]. Cells were cultured in glucose-free media and incubated in 50 µM 2-NBDG for 45 min. Subsequently, cells were washed, and fluorescence was measured using flow cytometry.

### Flow cytometry analysis

Propidium iodide (PI; Sigma-Aldrich, St. Louis, MO) was used to stain the cancer cells and then the cancer cells were subject to flow cytometry analysis as previously described [44]. All cells were starved of FBS for overnight as base line and then the cells were released into normal cell culture DMEM medium for 48 h. A total of 10,000–20,000 stained nuclei were collected for cell cycle analysis using the ModFit LT software (Verity Software House, Topsham, ME).

### Co-culture bronchial epithelial cells with feeder layer

Swiss 3T3-J2 mouse fibroblasts were grown in DMEM medium containing 1% penicillin/streptomycin and 10% fetal bovine serum. When the mouse fibroblasts cells reached 70% confluency, the cells were irradiated at 30 Gy (3000 rads) as feeder layer.

Bronchial epithelial cells were obtained by brushings (Bronchoscopy cytology brush, Cook Medical) and collected in normal saline on ice following an established protocol [45]. Bronchial brushings cells were centrifuged at 300 g for 5 min and applied on top of feeder layer and cultured in the presence of the ROCK inhibitor at a final concentration of 5 mM (Enzo Life Sciences, Farmingdale, NY).

### Western blotting

Total protein extracts and Western blot (WB) analysis were performed as in previous studies [41]. Primary antibodies dilutions were performed as the following: GLS1 at 1:1,000 (#7485-1 Epitomics, Burlin-game, CA), xCT at 1: 1,000 (#12691, Cell Signaling Technology, Danvers, MA), Cyclin D1 at 1: 1,000 (#2978, Cell Signaling Technology), BCL-xL at 1: 1,000 (#2762, Cell Signaling Technology), MYC at 1: 1,000 (#13987, Cell Signaling Technology), and Actin at 1: 5,000 (#3700, Cell Signaling Technology, MA).

### Immunohistochemistry

Immunohistochemical staining for xCT protein using anti-xCT (#12691, Cell Signaling Technology, Danvers, MA) and scoring was conducted as previously described [41]. Briefly, the final staining score was calculated by multiplying the staining distribution score (no staining = 0; 0.1, staining of 1%-9% of cells = 0.1; 10%–49% = 0.5 and if > 50% of cells, socre = 1) by the staining intensity score (no staining = 0; weak = 1; moderate = 2; strong = 3). The median value of all the final staining scores was used for distinguishing xCT_high tumors from xCT_low tumors.

### Mouse xenograft study

Stably transfected H520 cells (5 × 10^6^) with either xCT_KD or control construct were injected into the flanks of ten female nude mice-foxn1nu (Harlan Laboratories, Indianapolis, IN, USA). Tumor growth was monitored up to 4 weeks and measured with calipers. Immunohistochemistry for xCT, cleaved caspase 3, and Ki-67 was performed according to a previous protocol [41]. Images were taken and analyzed with Olympus BX41TF microscope and Olympus cellSens software.

### Statistics

UCSC Cancer Genomics Browser [46] was used to download TCGA lung cancer mRNA expression dataset (IllumninaHiSeq_RNASeqV2). The Student’s t-test was used to compare the level of xCT mRNA expression in lung tumors vs. normal tissues. The association between xCT protein expression and overall survival after adjusting for age, gender, smoking status, and the stage was assessed using Cox proportional hazards regression model. The survival difference was analyzed by the log-rank test based on median xCT expression using the Kaplan-Meier method. Statistical analyses for the glutamine uptake kinetics and cell growth assays were conducted with GraphPad Prism (GraphPad Software, La Jolla, CA). All results were demonstrated as the mean ± SD which were performed at least three independent measurements (as indicated in figure legends). Individual p-values were reported in the figures with values of *p* < 0.05 considered as statistically significant.

## Electronic supplementary material

Supplementary Legends

Supplementary Figures
