# Supplementary material for: xCT (SLC7A11)-mediated metabolic reprogramming promotes non-small cell lung cancer progression
Source: Oncogene. 2018 May 23;37(36):5007–19. doi: 10.1038/s41388-018-0307-z (PMC6127081; doi:10.1038/s41388-018-0307-z)
Supplement: Supplementary file 1 — Supplementary Legends [file 41388_2018_307_MOESM1_ESM.pdf]

Supplementary Figure 1 **xCT suppression by shRNA inhibited proliferation, invasion, and colony formation in NSCLC cells*.***

(**A**) The expression of xCT was successfully suppressed by ShRNA in A549, H520, HCC15 and HCC95 NSCLC cells. (**B**) Cell proliferation assays revealed significant growth inhibition induced by xCT knockdown in A549 (n=4). (**C**) Cell proliferation assays revealed significant growth inhibition induced by xCT knockdown in HCC15 (n=4). (**D**) Cell proliferation assays revealed significant growth inhibition induced by xCT knockdown in HCC95 (n=4). (**E**) Knockdown of xCT significantly reduced the A549 cell invasion compared to the A549_Ctrl by invasion assay (n=4). (**F**) Knockdown of xCT significantly reduced the HCC15 cell invasion compared to the HCC15_Ctrl by invasion assay (n=4). (**G**) Knockdown of xCT significantly reduced the HCC95 cell invasion compared to the HCC95_Ctrl by invasion assay (n=4). Colony-formation assays showed that xCT knockdown significant reduced the capacity of A549 (**H**) and H520 (**I**) to form colonies in soft agar (n=3).

Supplementary Figure 2 **Suppression of xCT genetically or pharmaceutically inhibited tumor formation *in vivo.***

(**A**) Representative H&E staining, immunohistochemistry staining of, Ki67, cleaved Caspase 3 and xCT in tumor formed by H520_Ctrl and H520_xCT_KD. (**B**) The relative signal intensity of Ki67 and cleaved caspase 3 (n=3). Glutamine deprivation assays showed that xCT knockdown reduced the glutamine dependency in HCC15 (**C**) and HCC95 (**D**) in 72 hours (n = 4). (**E**) SASP treatment protocol in vivo. (**F**) The relative signal intensity of Ki67 and cleaved caspase 3 (n=3).

Supplementary Figure 3 **Knock down of xCT reduced the glycolysis and GSH/GSSG ratio *in vitro*.**

(**A**) The knockdown of xCT significantly decreased the glucose consumption in A549, HCC15 and HCC95 cells (n=4). (**B**) The knockdown of xCT significantly decreased the lactate production in A549 and HCC95 cells (n=4). (**C**) The knockdown of xCT significantly decreased the GSH/GSSG ratio in A549, HCC15, and HCC95 cells (n=6).

Supplementary Figure 4 **The overexpression of xCT promoted more reductive intracellular condition *in vitro*.** (**A**) The overexpression of xCT promoted more reductive intracellular condition in the 16HBE cells (n=6). (**B**) The overexpression of xCT reduces ROS in 16HBE cells (n=6). The overexpression of xCT induced the glucose uptake in 16HBE cells (n=3) (**C**) and in BEAS2B cells (n=3) (**D**). The expression of xCT was induced by the treatment of cigarette smoking condensate (40 µg/ml, 72 hours) in BEAS2B cells (**E**) and in primary bronchial epithelial cells (40 µg/ml, 14 days) (**F**). The overexpression of xCT significant increased the sensitivity of SASP (1mM, 48 hours) in 16HBE and BEAS2B cells (**G**).

Supplementary Figure 5 **The expression of xCT determined the cellular glutamate secretion and migration *in vitro*.** (**A**) The knockdown of xCT increased the intracellular level of glutamate in H520 cells (n=6). (**B**) The overexpression of xCT significantly reduced the intracellular level of glutamate in BEAS2B cells (n=6). (**C**) The overexpression of xCT promote the cell migration compared with the control (left panel) in BEAS2B cells; The overexpression of xCT improve the inhibitory effects of SASP on the cell migration compared with the control (right panel) in BEAS2B cells (n=3)

Supplementary figure 6 **Images of Western blot film of Fig 1D**
Supplementary figure 7 **Images of Western blot film of Fig 3A**
Supplementary Figure 8 **Images of Western blot film of Fig 4G**
Supplementary Figure 9 **Images of Western blot film of Supplementary Fig 1A**
Supplementary Figure 10 **Band densitometries of Fig 1D and 3A**
Supplementary Figure 11 **Images of Western blot film of Supplementary Fig 4E**
Supplementary Figure 12 **Images of Western blot film of Supplementary Fig 4F**
