# Supplementary material for: xCT (SLC7A11)-mediated metabolic reprogramming promotes non-small cell lung cancer progression
Source: Oncogene. 2018 May 23;37(36):5007–19. doi: 10.1038/s41388-018-0307-z (PMC6127081; doi:10.1038/s41388-018-0307-z)

Supplementary Figure 1.

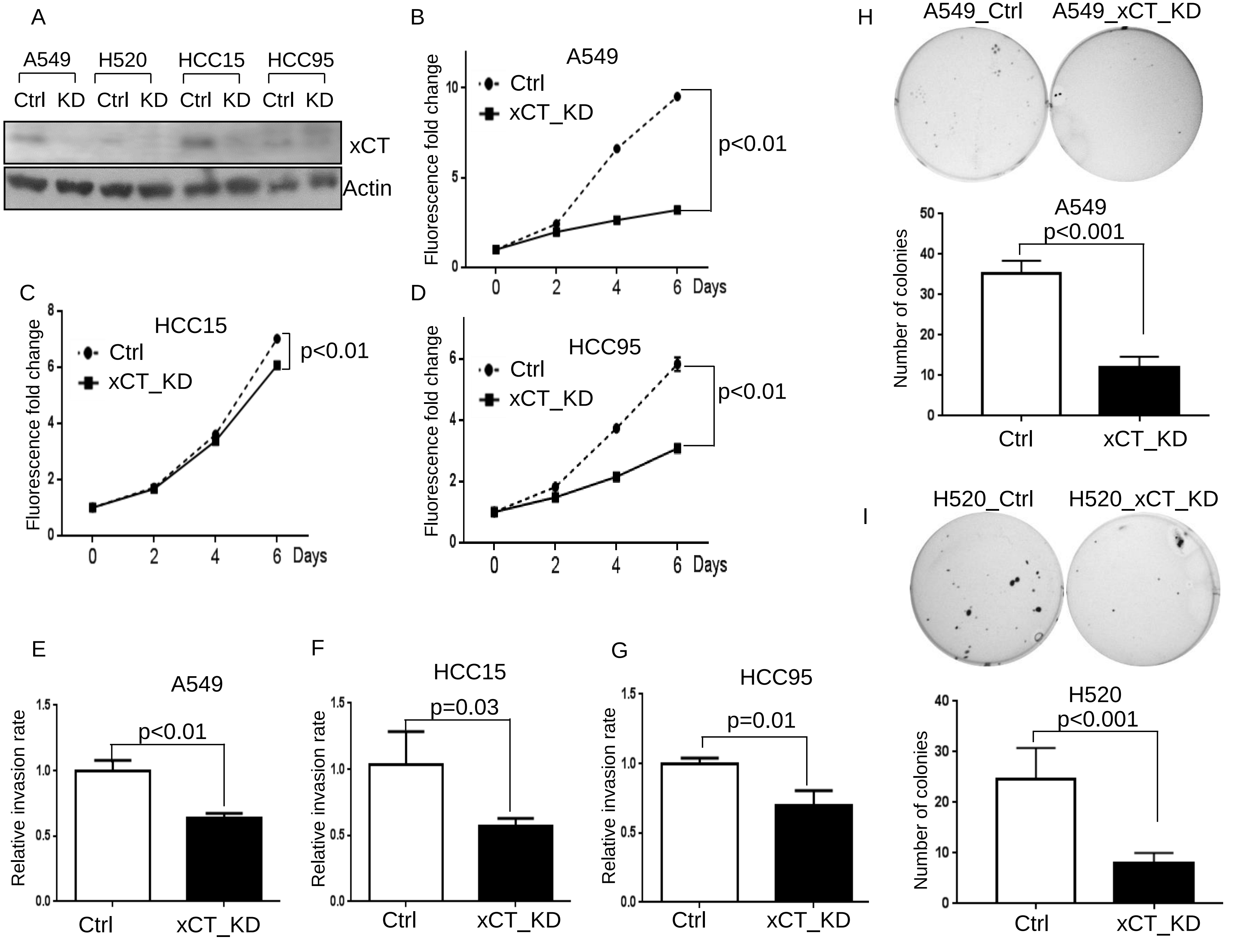

Supplementary Figure 2.

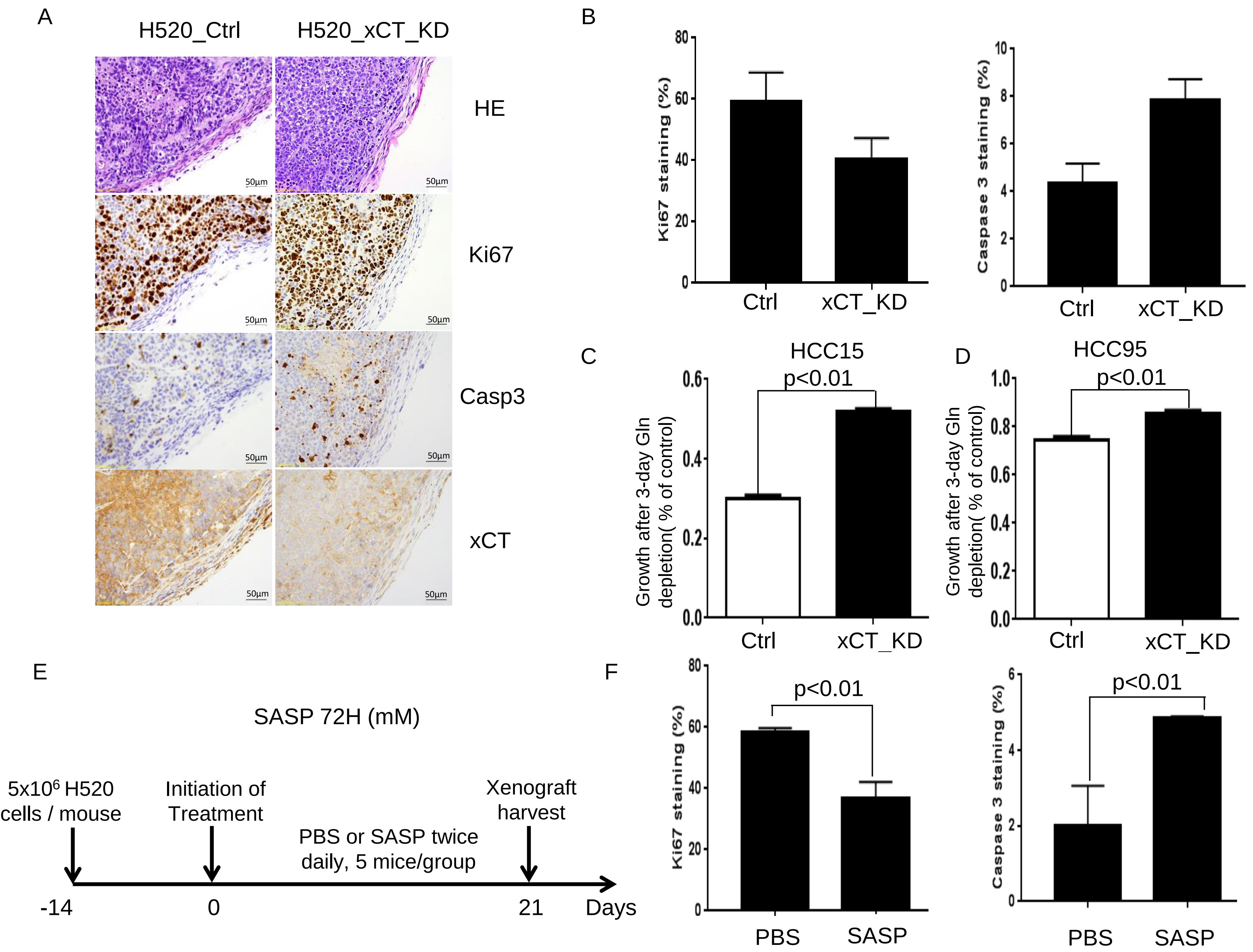

Supplementary Figure 3.

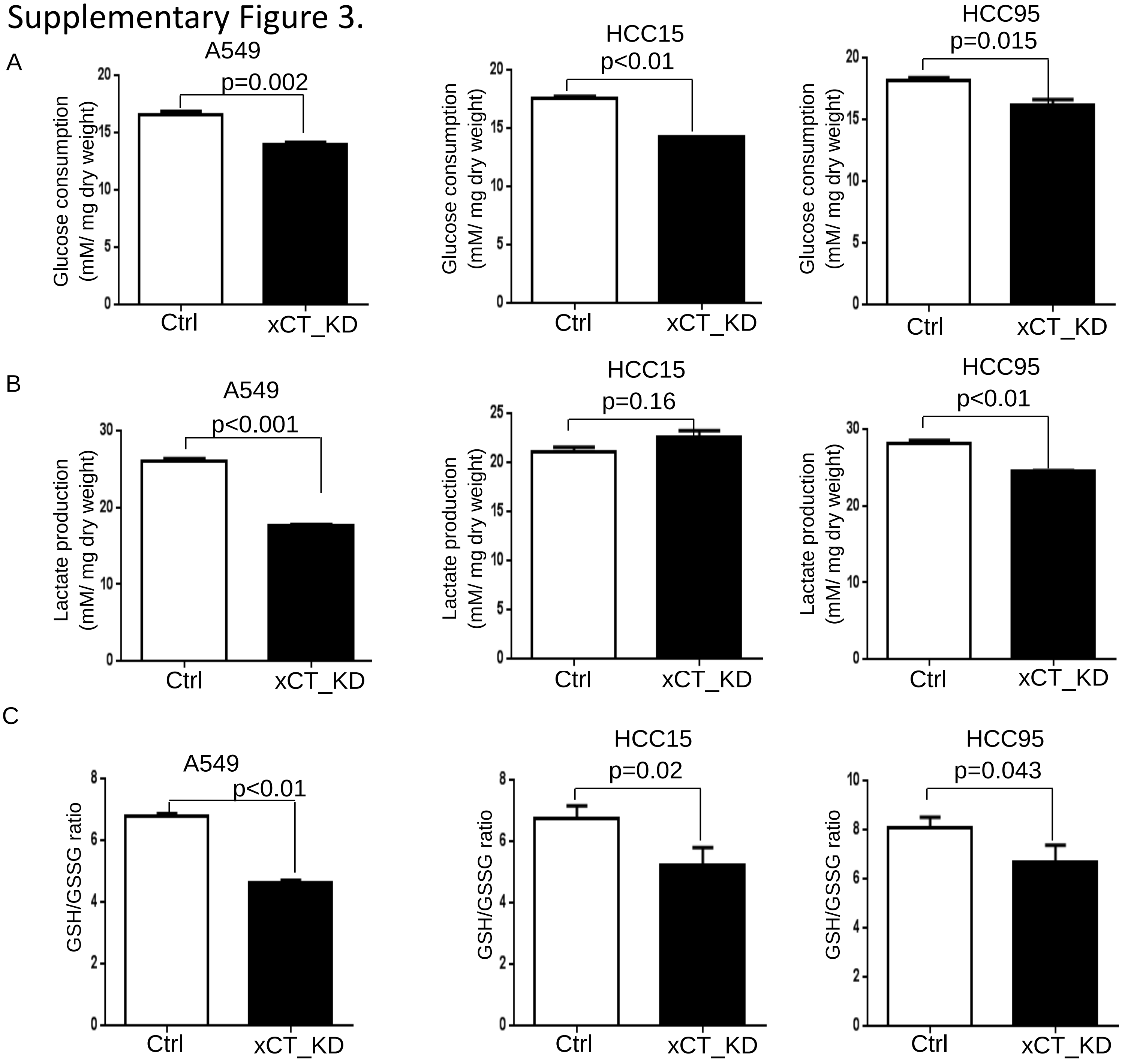

Supplementary Figure 4.

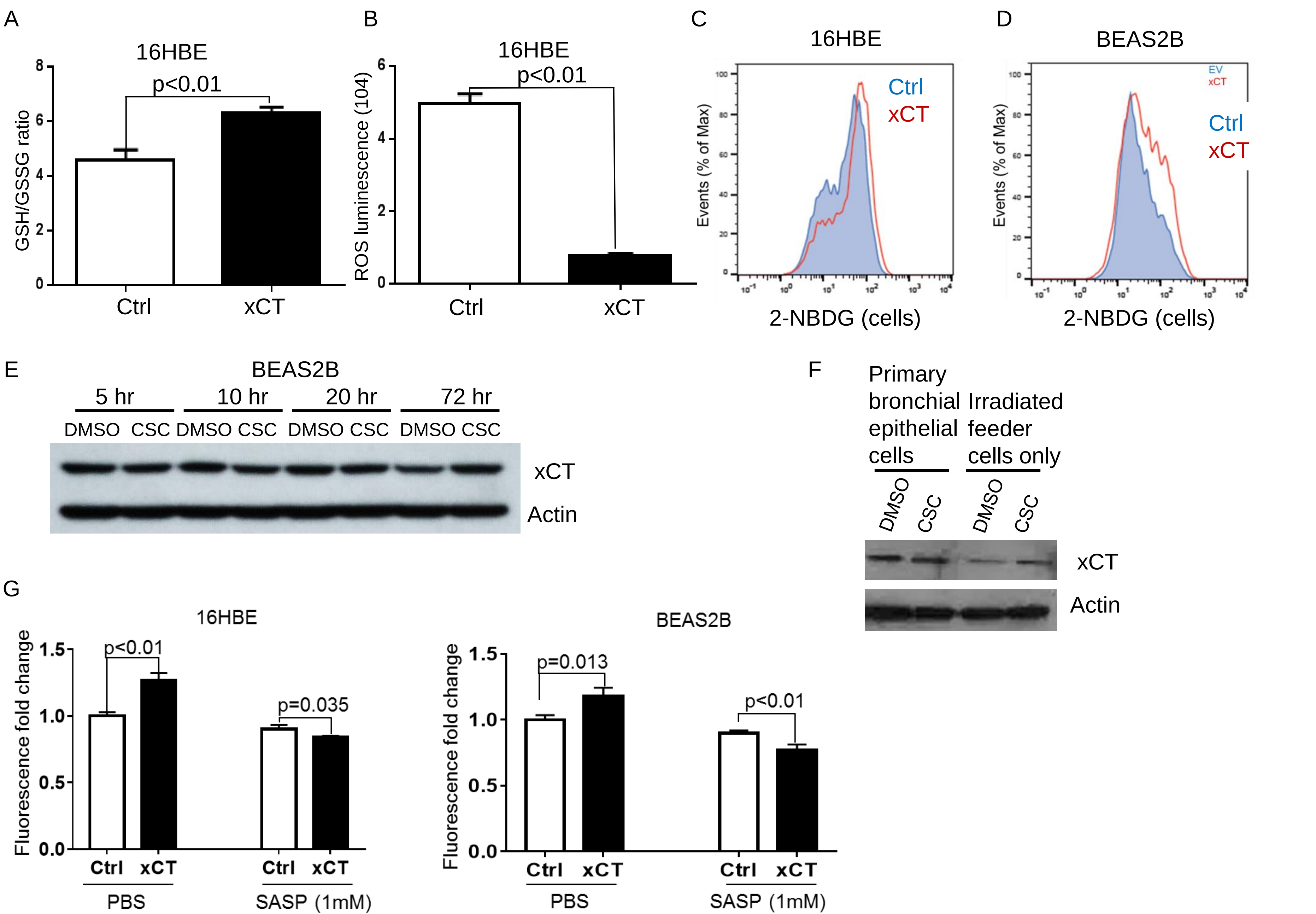

Supplementary Figure 5.

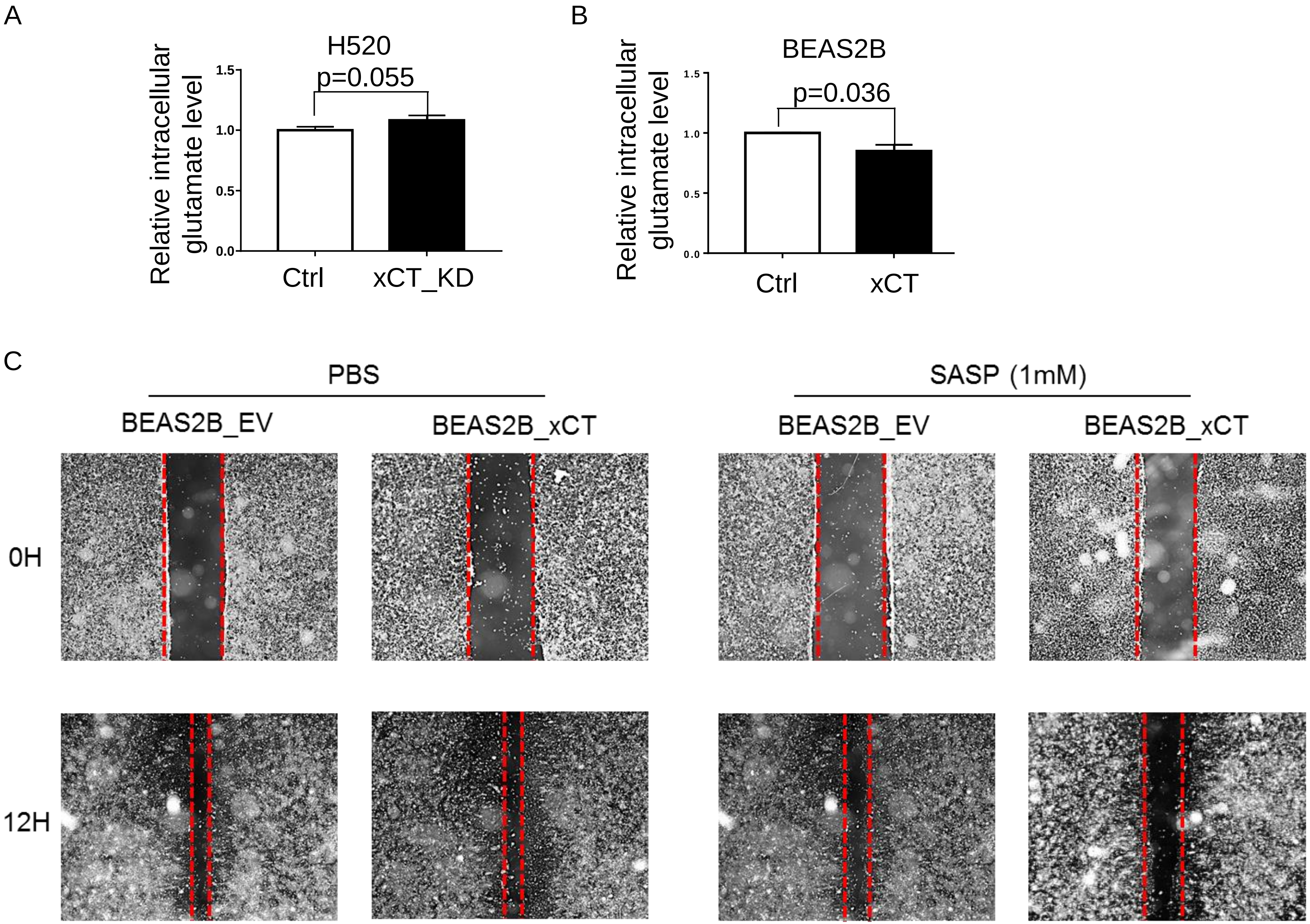

Supplementary Figure 6.

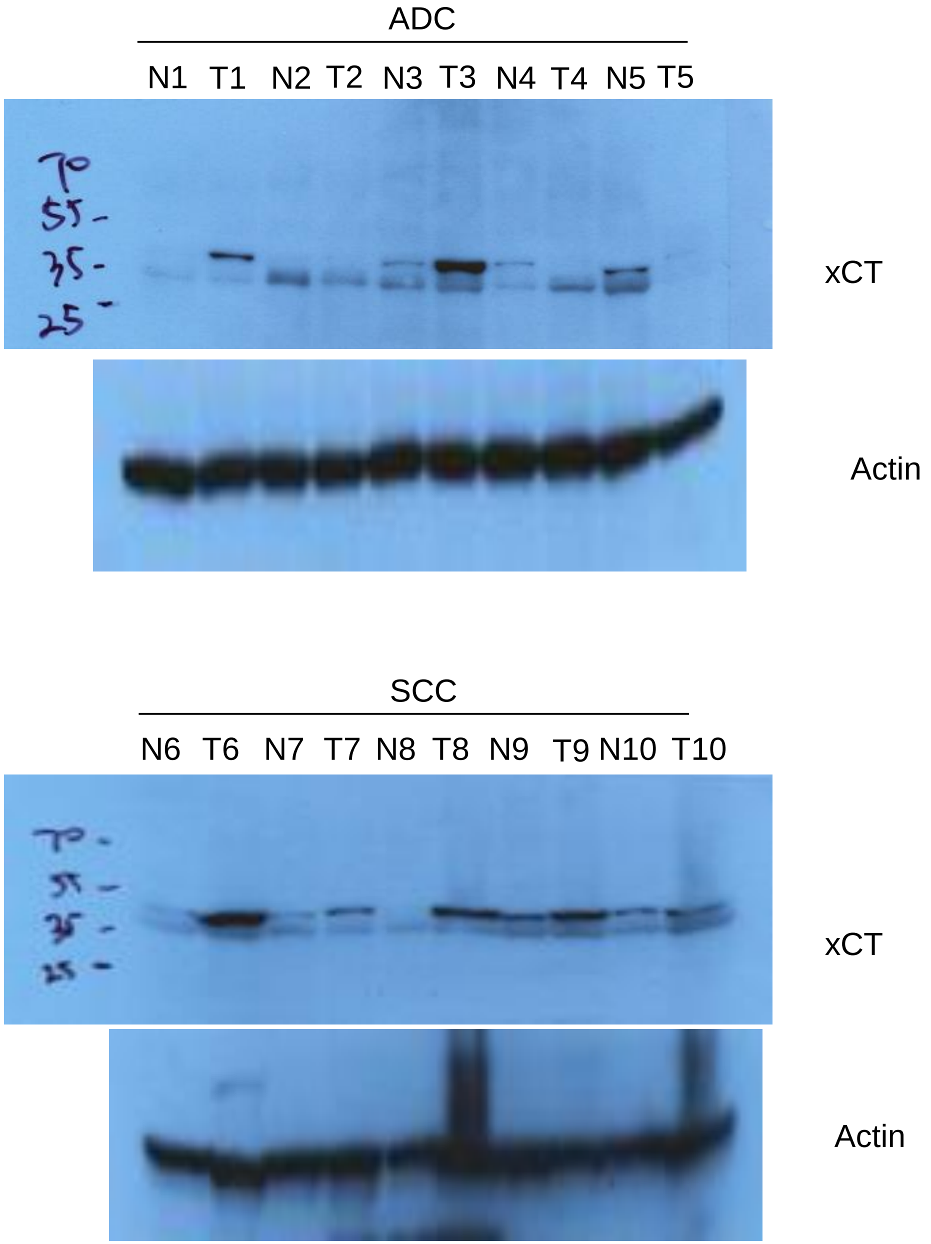

Supplementary Figure 7.

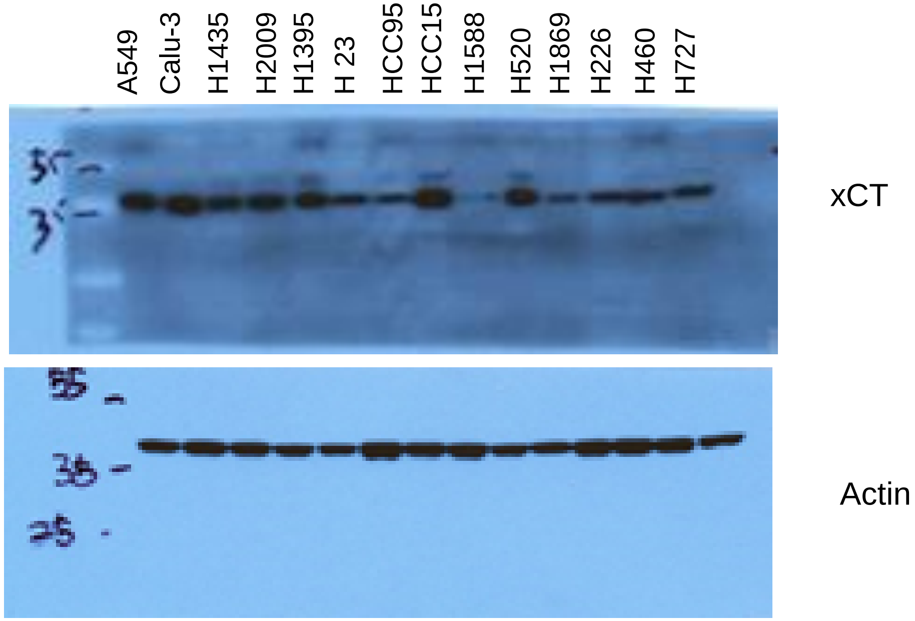

Supplementary Figure 8.

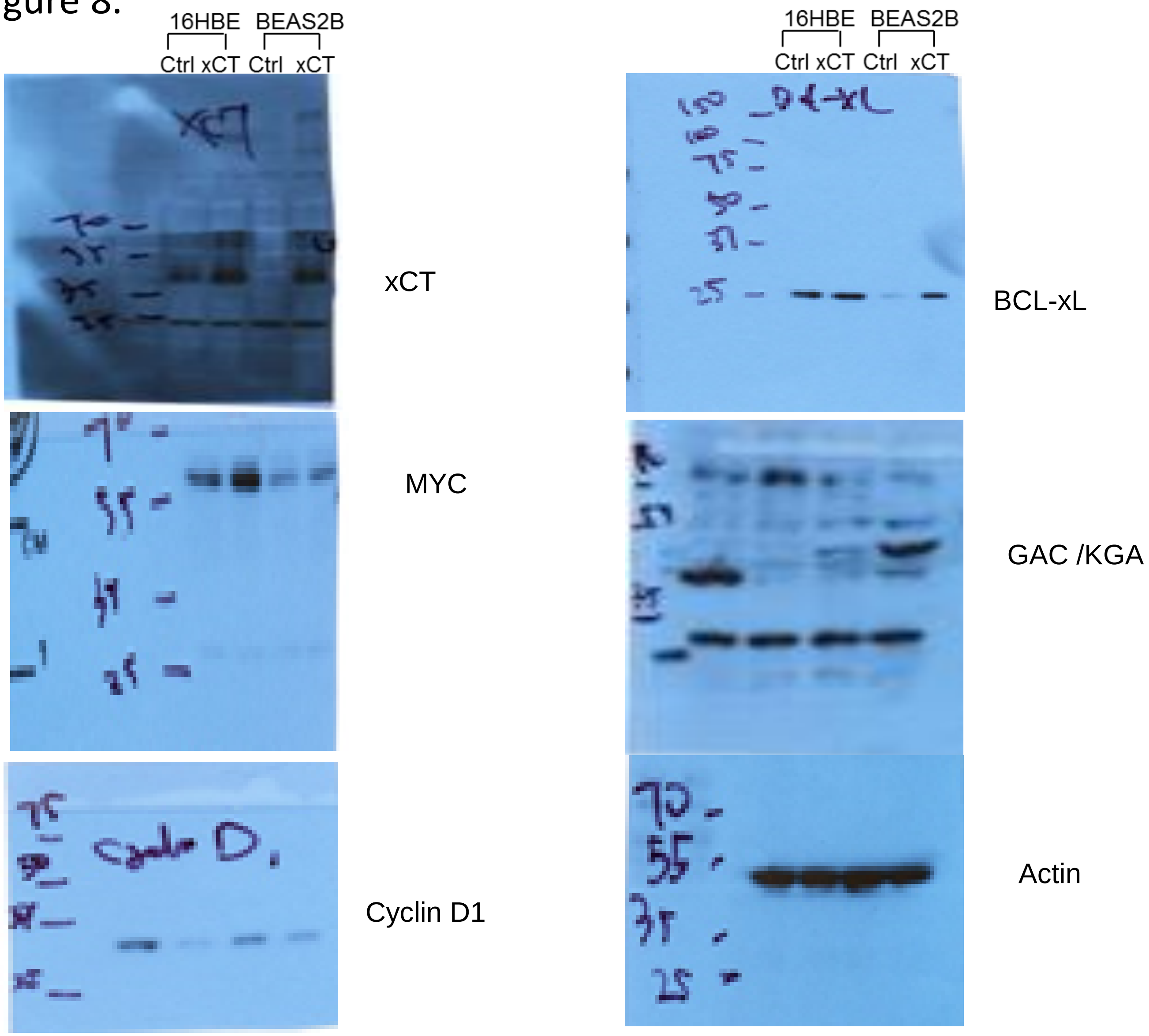

Supplementary Figure 9.

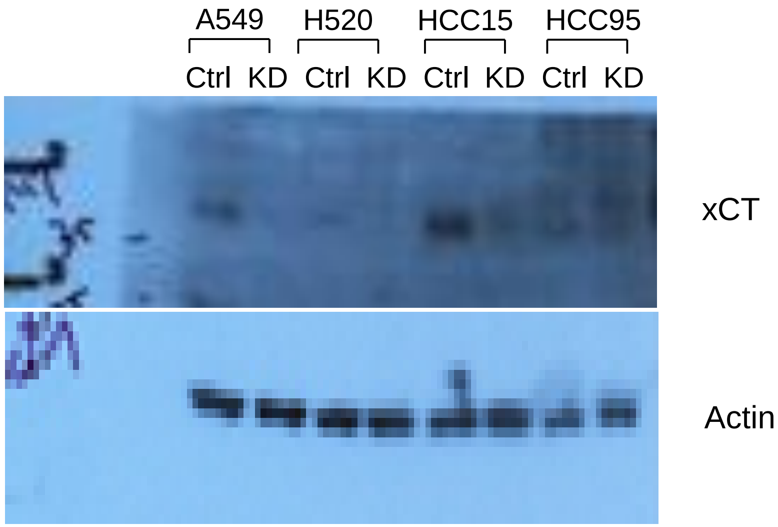

Supplementary Figure 10.

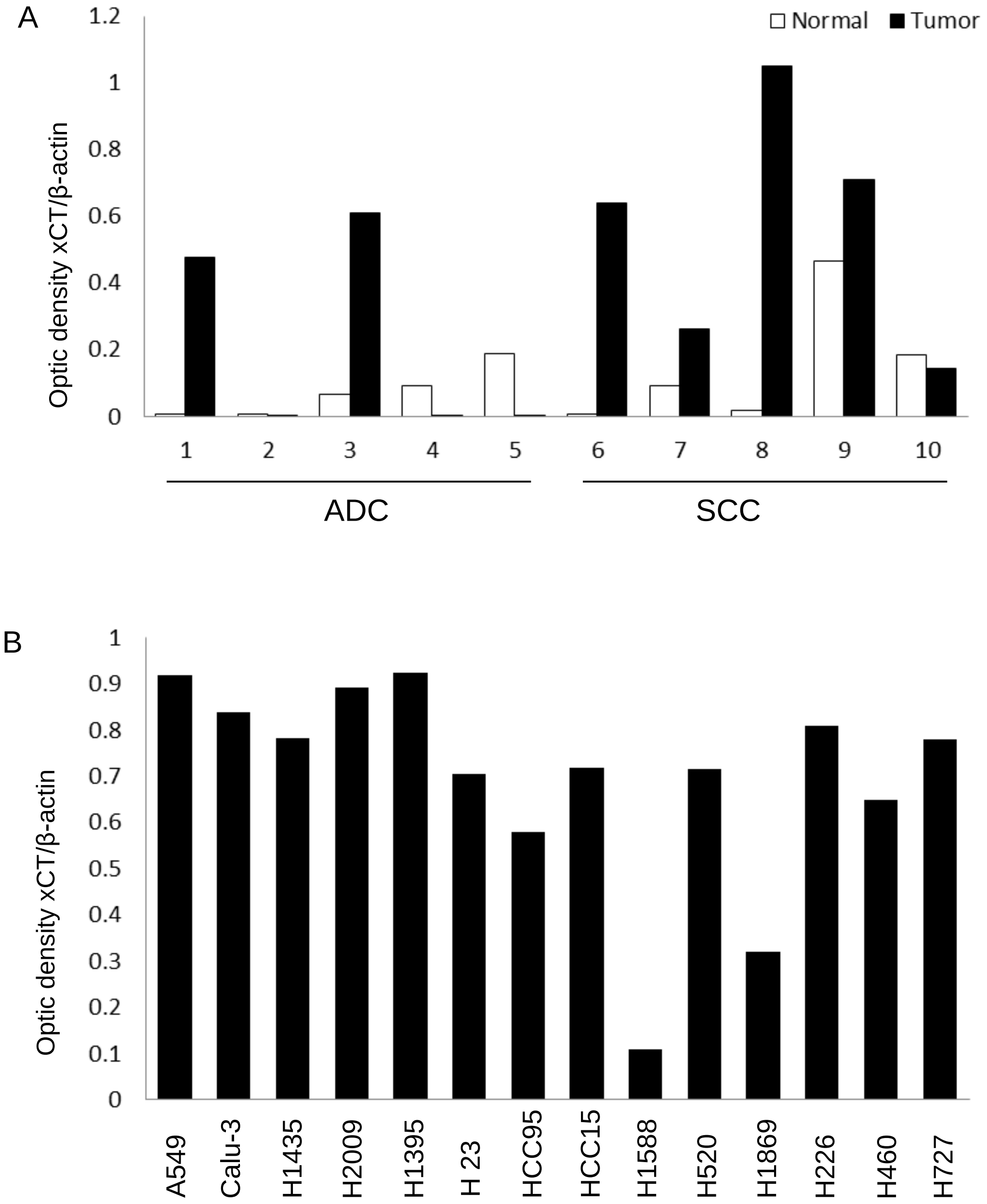

Supplementary Figure 11.

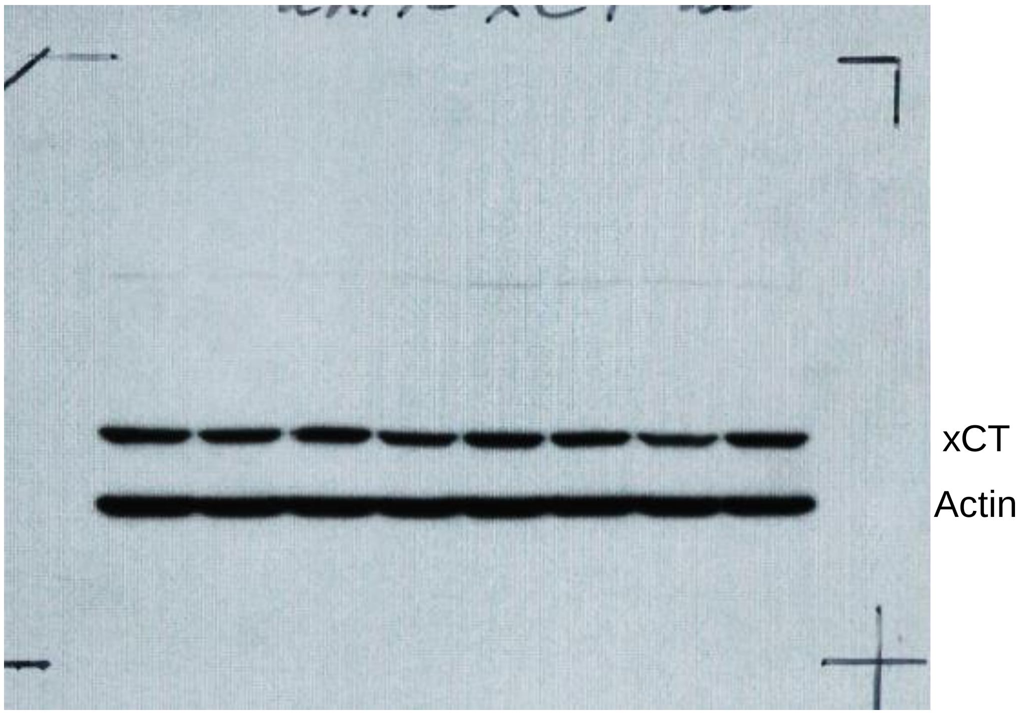

Supplementary Figure 12.

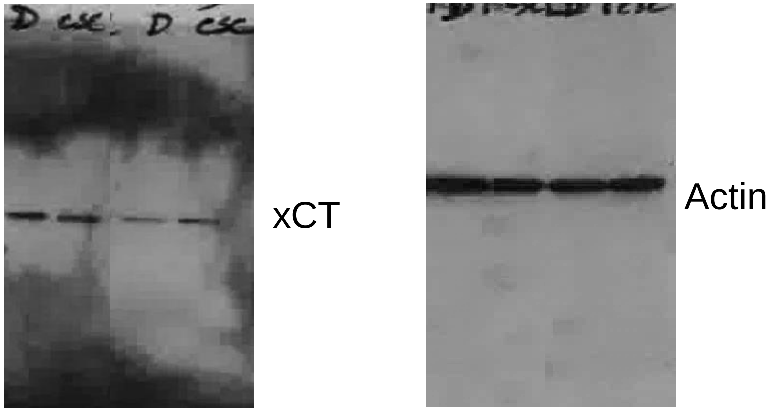

Supplement: Supplementary file 2 — Supplementary Figures [file 41388_2018_307_MOESM2_ESM.pdf]
